# Skin lesion segmentation with a multiscale input fusion U-Net incorporating Res2-SE and pyramid dilated convolution

**DOI:** 10.1038/s41598-025-92447-1

**Published:** 2025-03-07

**Authors:** Zhihui Liu, Jie Hu, Xulu Gong, Fuzhong Li

**Affiliations:** 1 College of Software, Shanxi Agricultural University, Taigu, Mingxing, 030801 China; 2 College of Agricultural Engineering, Shanxi Agricultural University, Taigu, Mingxing, 030801 China

**Keywords:** Skin lesion segmentation, Deep learning, Multiscale input fusion, Squeeze and excitation, Pyramid dilated convolution, Residual structures, Biological techniques, Computational biology and bioinformatics

## Abstract

Skin lesion segmentation is crucial for identifying and diagnosing skin diseases. Accurate segmentation aids in identifying and localizing diseases, monitoring morphological changes, and extracting features for further diagnosis, especially in the early detection of skin cancer. This task is challenging due to the irregularity of skin lesions in dermatoscopic images, significant color variations, boundary blurring, and other complexities. Artifacts like hairs, blood vessels, and air bubbles further complicate automatic segmentation. Inspired by U-Net and its variants, this paper proposes a Multiscale Input Fusion Residual Attention Pyramid Convolution Network (MRP-UNet) for dermoscopic image segmentation. MRP-UNet includes three modules: the Multiscale Input Fusion Module (MIF), Res2-SE Module, and Pyramid Dilated Convolution Module (PDC). The MIF module processes lesions of different sizes and morphologies by fusing input information from various scales. The Res2-SE module integrates Res2Net and SE mechanisms to enhance multi-scale feature extraction. The PDC module captures image information at different receptive fields through pyramid dilated convolution, improving segmentation accuracy. Experiments on ISIC 2016, ISIC 2017, ISIC 2018, PH2, and HAM10000 datasets show that MRP-UNet outperforms other methods. Ablation studies confirm the effectiveness of its main modules. Both quantitative and qualitative analyses demonstrate MRP-UNet’s superiority over state-of-the-art methods. MRP-UNet enhances skin lesion segmentation by combining multiscale fusion, residual attention, and pyramid dilated convolution. It achieves higher accuracy across multiple datasets, showing promise for early skin disease diagnosis and improved patient outcomes.

## Introduction

Skin diseases account for a significant proportion of the global disease burden^1,2^. Accurate lesion segmentation is crucial for the early diagnosis of skin cancer and improving patient survival rates. Manual screening of dermoscopy images is time-consuming and tedious. It is also subject to observer variability, a challenge worsened by the shortage of dermatologists^3^. Lesion segmentation is the initial step in identifying and diagnosing skin diseases. Deep learning-based segmentation algorithms can automatically delineate lesion areas in medical images. This leads to more efficient identification and evaluation of clinically relevant features, improving both treatment outcomes and diagnostic accuracy^4,5^. This paper focuses on skin lesion segmentation to assist researchers in developing image analysis tools for the automated diagnosis of skin lesions using dermoscopic images. Dermoscopy is a non-invasive imaging technique^6^. It captures magnified, well-lit images of localized skin areas while reducing surface reflections, thus enhancing lesion clarity. Segmentation algorithms classify each pixel as part of the lesion or the background. This helps identify regions of interest for further analysis, ultimately improving diagnostic efficiency. Skin lesion segmentation plays a critical role in computer-aided diagnosis (CAD)^7^. This study aims to advance the automatic diagnosis of melanoma from dermoscopic images.

**Fig. 1:**
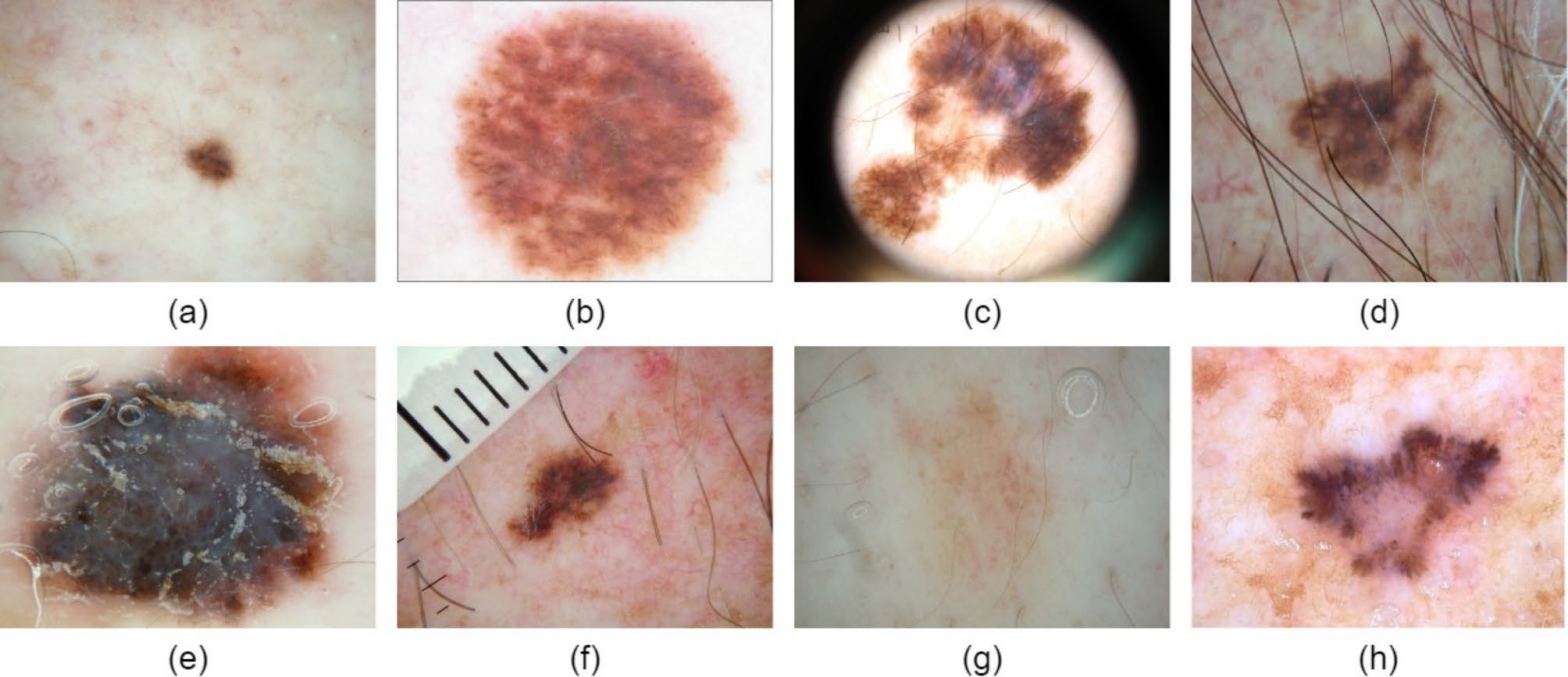
Examples of skin lesions with diverse characteristics in public dataset: (**a**) small-sized skin lesions; (**b**) large-sized skin lesions; (**c**) irregularly shaped skin lesions; (**d**) skin lesions obscured by hairs; (**e**) skin lesions with air bubbles; (**f**) skin lesions with vessels; (**g**) skin lesions with low contrast against the background; (**h**) skin lesions with varying color.

The lesion area has low contrast with normal areas, making it difficult to accurately segment the lesion^8^. Moreover, different skin conditions (e.g. skin color, hair, or blood vessels) can produce very different melanoma appearance^9^ Automatic segmentation of lesion areas from dermoscopic images is a challenging task. As shown in Fig. 1, lesions in these images vary significantly in size and have irregular borders. They also exhibit non-uniform color distribution and low contrast with surrounding normal skin, which makes automated skin lesion segmentation extremely difficult. Additionally, artifacts such as hairs, blood vessels, and air bubbles in the images can interfere with the system’s ability to segment lesions automatically^10^. These factors further increase the complexity of the segmentation process.

**Fig. 2:**
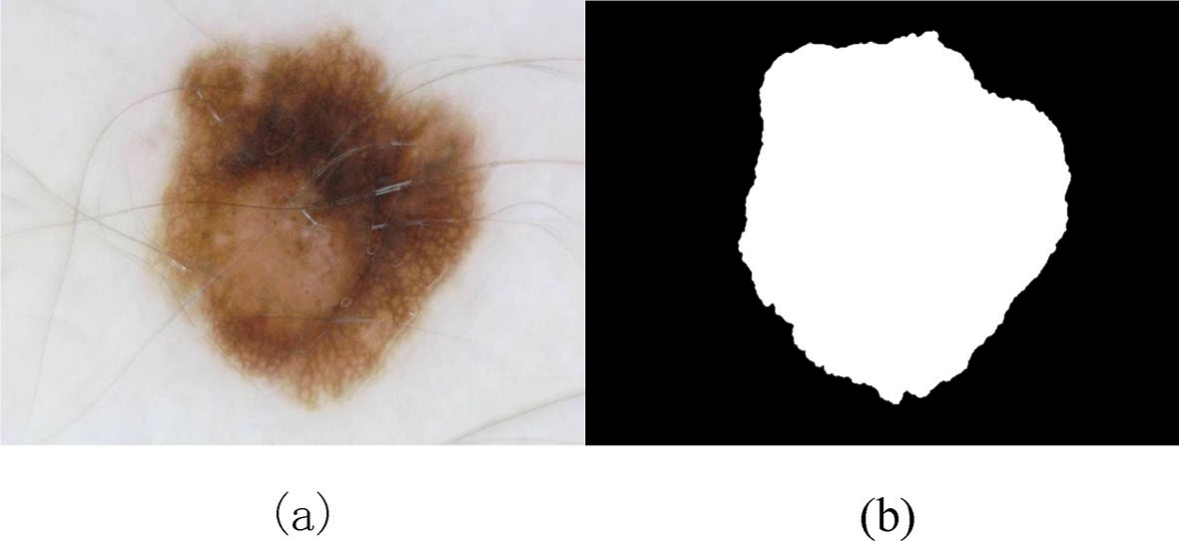
Illustration of a skin lesion image and its annotated ground truth mask(From ISIC 2018 dataset). (**a**) Original dermoscopy image (**b**) Corresponding ground truth mask.

Semantic segmentation involves assigning an appropriate class label to each pixel to distinguish lesions from the surrounding skin. Figure 2a shows an example of an original skin lesion image. Figure 2b illustrates the “ground truth” segmentation image. The task of semantic segmentation is to generate a segmentation map similar to Fig. 2b from an original input image like Fig. 2a.

In recent years, deep learning methods have made breakthrough progress in the field of skin lesion segmentation. The fully convolutional network (FCN)^11^ has achieved satisfactory results in segmentation tasks. However, FCN based methods^12–14^ usually face the following limitations: as the network deepens, the resolution gradually decreases, leading to the loss of spatial information; the receptive field of convolution is limited and cannot model long-distance dependencies, resulting in insufficient contextual information; the feature representation of boundary region pixels is weak, causing a large number of semantic segmentation errors to be concentrated in the boundary regions. Ronneberger et al. proposed U-Net for biomedical image segmentation^15^. The encoder extracts features from the image input, and the decoder restores the encoded features and outputs the final result. Skip connections concatenate corresponding encoder-decoder pairs. Many U-Net-based vascular segmentation architectures have been proposed. CE-Net was proposed to capture more high-level information and preserve spatial information^16^. It improves the network’s performance in lesion segmentation by introducing useful strategies in the encoder-decoder structure. A context extraction module is added between the encoder and decoder at the highest dimension level to enhance the network’s global information. However, the down-sampling operation of this model causes the loss of spatial information of small vessels in retinal images, which ultimately cannot be recovered by skip connections or up-sampling operations. Wang et al. designed a new network architecture called SAR-U-Net^17^. It uses SE and Atrous Spatial Pyramid Pooling (ASPP) to achieve accurate and reliable image segmentation. This network adaptively extracts image features, suppresses irrelevant regions, and highlights features specific to the segmentation task. Lei et al. proposed a Generative Adversarial Network (GAN) that uses U-Net segmentation module with skip connections and dense convolutions to generate deep representations^18^. The dual discriminator (DD) module focuses on the boundary differences and the contextual environment of the target object in the generated segmentation mask. Yin et al. proposed a multi-scale fusion module that plays a crucial role in enabling the network to detect images of different scales and enhancing the network’s ability to capture complex details^19^.

Although many U-Net-based variant methods have achieved remarkable results, due to their weak feature extraction capabilities and insufficient utilization of skip connection modules, many methods still fail to handle challenging tasks well (such as low contrast with the background, irregular and blurry boundaries, small lesion areas, and hair occlusion). Inspired by the aforementioned methods, to address these issues, this paper proposes a Multiscale Input Fusion Residual Attention Pyramid Convolution Network (MRP-UNet) for effective lesion segmentation in dermoscopic images. Specifically, it consists of three modules: the Multiscale Input Fusion Module (MIF), the Res2-SE Module, and the Pyramid Dilated Convolution Module (PDC). MIF enhances the detection of skin lesions at various scales. This is achieved by constructing multiscale inputs using image pyramids and integrating these inputs with skip connections through a residual structure. The Res2-SE Module combines Res2Net and Squeeze-and-Excitation (SE) attention mechanisms to focus on important regions, replacing traditional 3 × 3 convolutions to improve feature extraction and segmentation accuracy. PDC utilizes dilated convolutions with varying dilation rates to expand the receptive field, capturing more contextual information and providing a comprehensive feature representation between the encoder and decoder.

To summarize, this paper includes the following main contributions:

1. Multiscale Input Fusion Module (MIF): Enhances detection of skin lesions at different scales by using image pyramids and integrating them with skip connections through a residual structure.
2. Res2-SE Module Integration: Combines Res2Net and SE attention mechanisms to replace the traditional 3 × 3 convolution in the U-Net encoder, focusing on important regions and enhancing feature extraction.
3. Pyramid Dilated Convolution Module (PDC): Expands the receptive field between the encoder and decoder using pyramid dilated convolutions, capturing more contextual information and improving feature representation.

The rest of the paper is organized as follows. Section "Related work" reviews related work and reviews recent research results that are closely related to our study. Section "Methodology" describes the proposed network architecture in detail. Section " Materials and experiments" presents materials and experiments. Section "Results" presents a comparative analysis of the results. Section "Limitations and future work" is limitations and future work. Finally, the paper concludes in Sect. "Conclusion".

## Related work

Some classical machine learning methods have been widely used in dermatology for lesion detection and segmentation, such as adaptive thresholding^20,21^, decision trees^22,23^, and support vector machines^24,25^. However, these methods require manual feature creation and are weak in feature extraction. In contrast, deep learning methods automatically extract and filter features, and segment the desired region without requiring manual manipulation or other preprocessing steps. Moreover, larger datasets lead to better results, as the depth of the network can be adjusted accordingly.

U-Net^15^ is commonly used for medical image segmentation. Its unique feature is the symmetric encoder-decoder structure. The encoder part consists of a convolutional layer and a pooling layer. These layers convert the image into a low-dimensional representation by extracting features from the input image and gradually reducing the size of the feature map, while removing irrelevant information. The decoder part mirrors the encoder and consists of a convolutional layer and an upsampling layer. These layers restore the low-resolution feature map to its original image size using inverse convolution. To compensate for the loss of spatial information during the downsampling process in the encoder stage, U-Net employs a jump-joining technique in each layer. The shallow feature maps in each upsampling layer of the decoder part are spliced with the corresponding shallow feature maps of the encoder part, thus retaining more original image information and effectively avoiding distortion of the segmentation results. Finally, the output of the upper layer is mapped to the desired segmentation class by a 1 × 1 convolutional kernel.

Afterward, various models based on the U-Net architecture have been proposed to further improve the performance of biomedical image segmentation. Lei et al. proposed a novel generative adversarial network (GAN) with dual discriminators for skin lesion segmentation^18^. The network utilizes a dense dilated convolutional U-Net architecture to enhance feature representation and improve segmentation accuracy, especially with limited training data. Wu et al. proposed ADAM model^26^. It extracts more comprehensive skin lesion features through global average pooling and pixel-level correlation modeling. Redundancy is reduced using spatial information weighting and adapted to lesions of different sizes based on dual encoder architecture and different expansion rates. Guo et al. proposed a lightweight SA-UNet network to address the problem of insufficient retinal vessel datasets^27^. This network does not require a large number of annotated samples and effectively utilizes existing data through data augmentation. It also introduces a spatial attention module and a structured dropout convolution block to effectively prevent overfitting. In recent years, attention mechanisms have gained prominence in computer vision (CV). They help neural networks adaptively focus on the most important parts of the input and ignore noise, thus enhancing the model’s feature extraction capabilities. The basic idea is to extract the global information of each channel from the output feature map of the network and use this information to adjust the weights of the channels. Attention Res-UNet improves on U-Net, which employs an attention mechanism that enables the network to learn in which regions to pay attention during segmentation, thus improving the accuracy of the model^28^. Wang et al. designed SAR-UNet, a new architecture based on U-Net^17^. It extracts the key features of the encoder through the SE module, suppresses the irrelevant regions, and uses the ASPP instead of the transformation and output layers to obtain multi-scale information. To address the problem of gradient vanishing, a residual structure replaces the traditional convolutional block. This improves liver segmentation accuracy while allowing for increased network depth. Res2Net is improved inside the residual block, and by establishing residual connections between the K input features inside the residual structure, multiple features of different scales can be obtained, thus improving the model’s ability to extract multi-scale features^29^. Res2Unet is used for retinal vessel segmentation^30^. The network employs a multi-scale strategy to extract blood vessels of different widths and integrates the strategy into the channel, greatly reducing parameters and computational resources. It also uses a channel attention mechanism to facilitate communication between channels and recalibrate the relationship of channel features.

Current methods for skin lesion segmentation, despite the results they have achieved, still have several key shortcomings. Traditional machine learning methods rely on manual feature creation and have limited feature extraction capabilities, making it difficult to handle complex skin lesion images. Deep learning methods such as U-Net and its improved models can extract features automatically though. However, there are still challenges in the integration of multi-scale information, the finiteness of the dataset and the overfitting problem, the ability of multi-scale feature extraction, the application of the attention mechanism, and the trade-off between the complexity of the network structure and the computational resources.

## Methodology

In this section, we introduce the key components of the MRP-UNet architecture: the Multiscale Input Fusion Module (MIF), the Res2-SE Module, and the Pyramid Dilated Convolution Module (PDC).

### Proposed network architecture

**Fig. 3:**
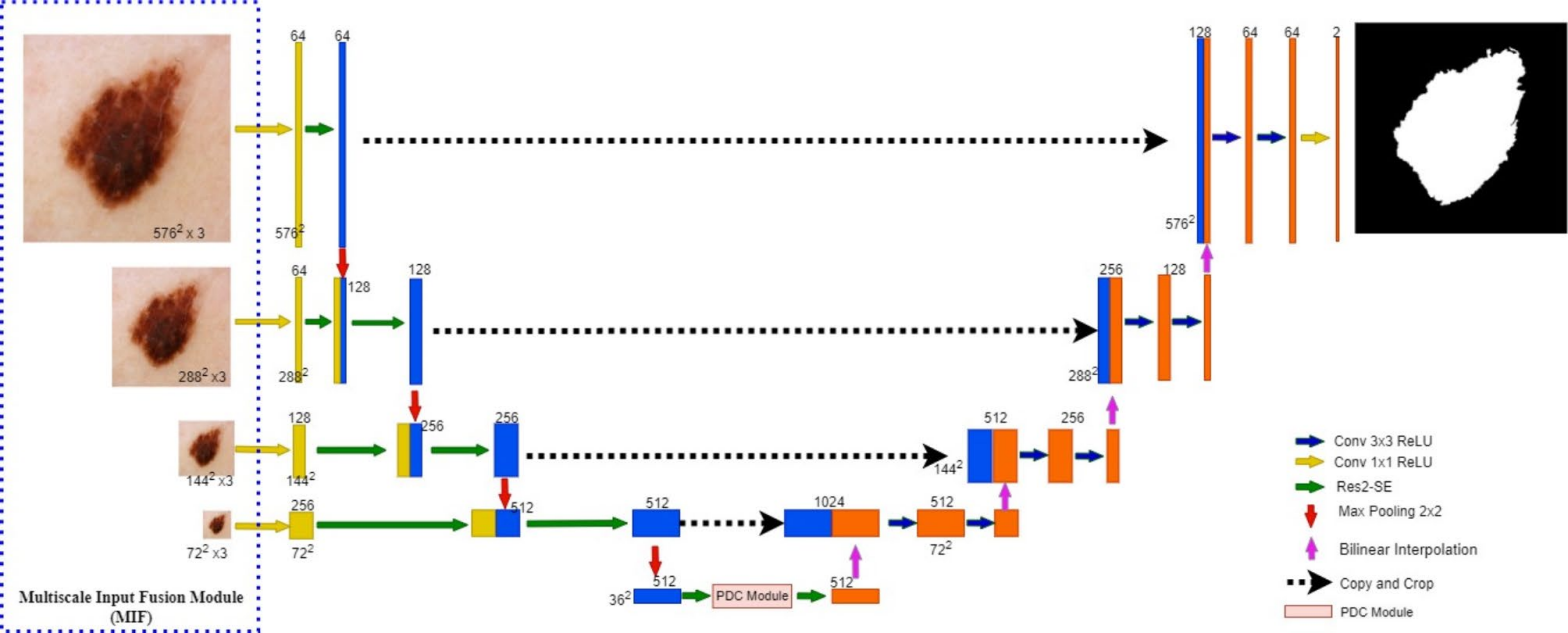
Network architecture of improved U-Net with multiscale input fusion module (*MIF*), Res2-SE module, and pyramid dilated convolution module (*PDC*).

We propose MRP-UNet, an enhanced version of the U-Net architecture tailored for image segmentation, as illustrated in Fig. 3. The figure provides a detailed illustration of the proposed MRP-UNet network structure, showcasing how the MIF, Res2-SE, and PDC modules are integrated to enhance segmentation performance. These modules work together to achieve the final segmentation task.

Once the MIF module generates multiscale features, these features are directly passed to the Res2-SE module. Through the collaboration between the Res2Net and SE modules, the network enhances its perception of features at different scales. This is followed by the PDC module, which captures global contextual information using convolutions with different dilation rates. During this process, the modules interact with each other through skip connections and multiscale feature interactions, ensuring the segmentation network’s continuity and accuracy. The specific roles of each part of the architecture are as follows:

1. Multiscale Input Fusion Module (MIF): The MIF module provides inputs at different scales, enabling the network to capture both large and small lesions. This directly enhances the network’s robustness when handling lesions of various sizes.
2. Res2-SE Module: This module is designed not only to expand the receptive field but also to optimize the weighting of channel features through the SE attention mechanism. This allows the network to focus more accurately on critical lesion areas during multiscale feature fusion, improving the efficiency of feature extraction. The Res2-SE module is introduced to address the limitations of the traditional U-Net structure in handling multiscale features. The Res2Net module expands the receptive field through residual connections, while the SE module adaptively enhances the weighting of key features, overcoming the traditional convolutional structure’s limitations when processing complex multiscale information.
3. Pyramid Dilated Convolution (PDC): This module not only expands the receptive field through different dilation rates but also ensures that the network captures both global and local contextual information through the design of parallel dilated convolution layers.

Through the multiscale input design of the MIF module, the network ensures robust detection of lesions of varying sizes. The Res2-SE module enhances feature extraction accuracy through multiscale feature processing and channel attention mechanisms. Finally, the introduction of the PDC module further expands the receptive field, effectively capturing both global and local contextual information. These modules collaborate to significantly improve the network’s performance in skin lesion segmentation tasks.

### Multiscale input fusion module

Multiscale input is an effective structure that has been shown to improve segmentation quality^19^. Multiscale input helps to detect skin lesion areas of different sizes. The Multiscale Input Fusion Module (MIF) proposed in this paper employs image pyramids to construct multiscale inputs, which enhances the network’s ability to perceive lesion regions of different scales. The MIF module is shown in the dashed box in Fig. 3.

First, an image pyramid is constructed for the input skin lesion images. The image pyramid includes the original size image and its different downsampling scales. In this paper, four sizes are used: 576 × 576 × 3,288 × 288 × 3,144 × 144 × 3 and 72 × 72 × 3, which represent the original image and its downsampled images of different scales, respectively. These images of different scales are used as inputs to the network respectively. Each scale image is passed through a layer of 1 × 1 convolution (yellow arrows) for channel number adjustment, and then proceeds to the next layer for further processing.

### Res2-SE module

**Fig. 4:**
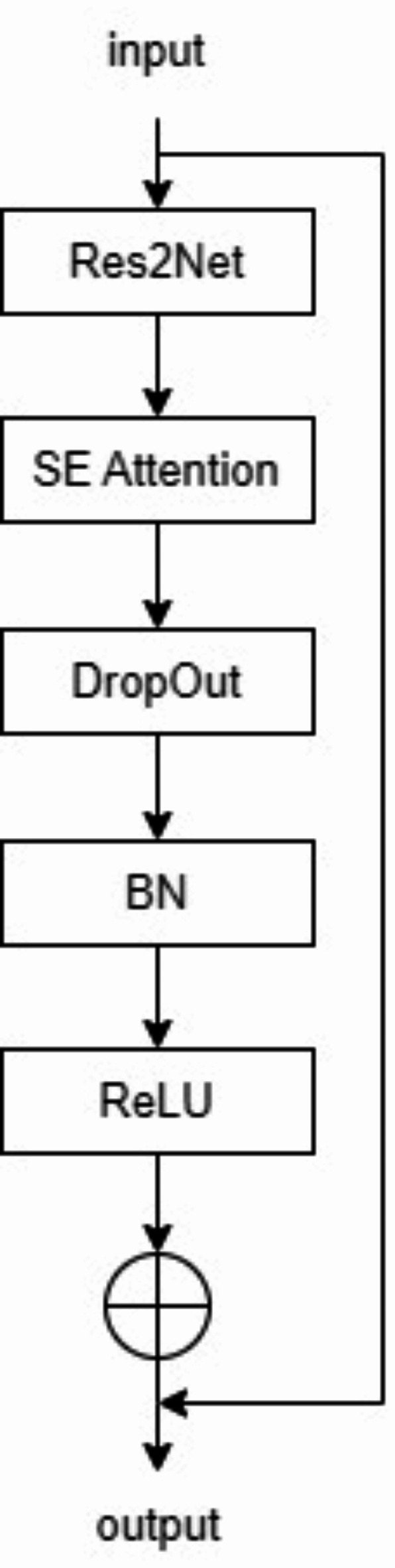
Structure of the proposed Res2-SE Net module.

Res2Net was first proposed in^29^. The residual structure inside the module allows for different sizes of receptive fields thus enabling the acquisition of multi-scale information in the input feature maps. Wang et al. introduced Squeeze-and-Excitation Networks to deal with the medical image segmentation problem^17^. The SE channel attention mechanism can weight the input information of different channels and suppress irrelevant information. Dropout is widely used in the field of medical image segmentation, which can accelerate network convergence and effectively prevent the overfitting problem in convolutional networks^27^. Inspired by the above method, this paper designs the Res2-SE structure, which replaces the original 3 × 3 convolution in the encoder part of the U-net. Feature extraction is performed by multiple 3 × 3 Res2-Net convolutions (green arrows) on each scale of the image separately. The Res2-SE structure is shown in Fig. 4. After the residual network Res2Net, the SE channel attention mechanism operation is performed and the SE is followed by Dropout, Batch Normalization (BN) layer, and Relu activation unit.

The design of the Res2-SE structure can help the network to better capture image features and improve the accuracy of image segmentation. Inside the Res2-SE structure, a Res2Net module^29^ is first executed. Then the SE channel attention module is used to evaluate the importance of different channels of the input feature map, so that the model can better access the feature information on different channels and improve the feature extraction ability of the model. Finally, the model is processed by dropout, batch normalization and activation function. The introduction of dropout and batch normalization layer can solve the problem of slow convergence or gradient explosion. The activation function increases the degree of nonlinearity of the network, so that the network can better extract the feature information in the input data.

#### Res2Net

**Fig. 5:**
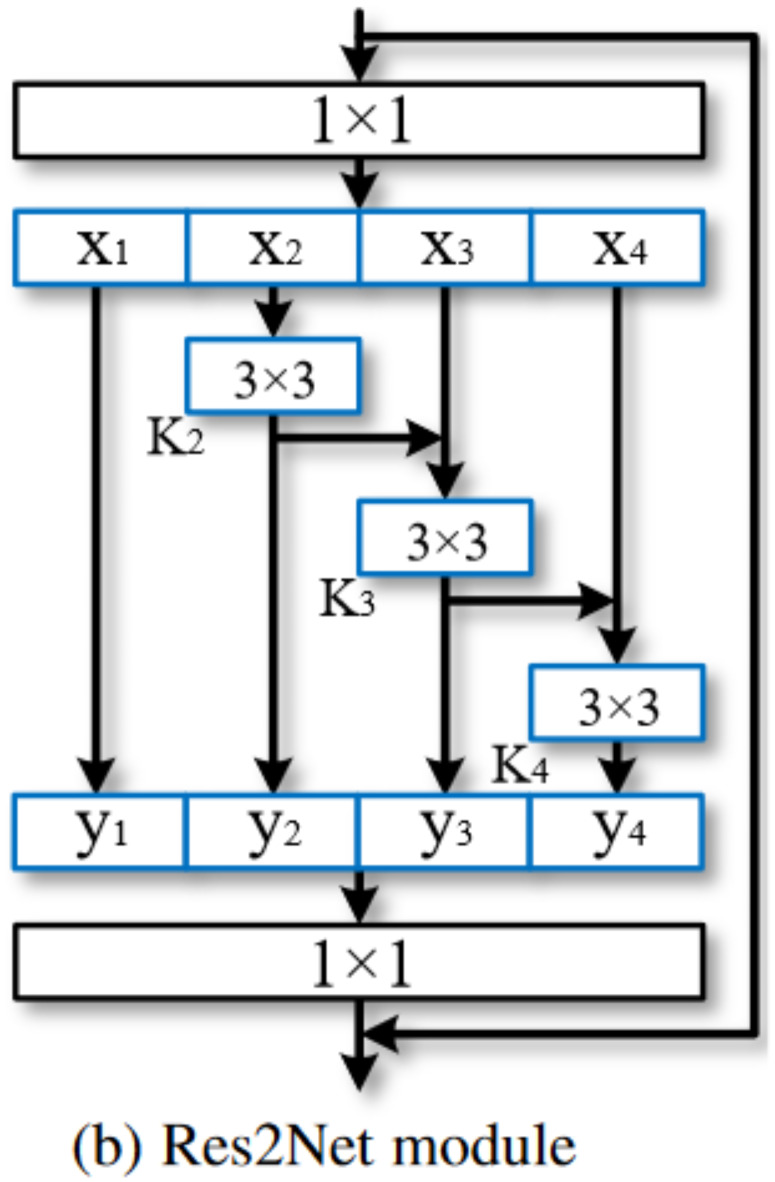
Res2Net module structure^29^.

Res2Net is a module obtained by improving the Bottleneck module in ResNet, and the module structure is shown in Fig. 5. Given an input feature map with $\:S$ channels, the information first undergoes a $\:1\times\:1$ convolution operation, dividing the feature map into n groups by channel, denoted as $\:{X}_{i}$, where$\:\:i\:\varepsilon \:\{ 1,\:2,\:...,\:n\}$. Each group’s channels are reduced to $\:S/n$. Starting from $\:{X}_{2}$ to $\:{X}_{n}$, each group undergoes a $\:3\times\:3$ convolution for feature extraction, represented by $\:{K}_{i}$. The output of the $\:i-th$ group’s 3 × 3 convolution is connected to the $\:(i+2)-th$ group using residual connection, continuing this pattern such that with increasing $\:i$, the number of convolution operations increases. Denote the output of channel $\:i$ as $\:{Y}_{i}$, can be expressed as:

$$\:{Y}_{i}=\left\{\begin{array}{c}\:\:\:{x}_{i}\:\:\:\:\:\:\:\:\:\:\:\:\:\:\:\:\:i=1\\\:{K}_{i}\left({x}_{i}\right)\:\:\:\:\:\:\:\:\:\:\:\:i=2\\\:{K}_{i}\left({x}_{i}+{y}_{i-1}\right)\:\:\:\:\:\:\:2<i\le\:n\end{array}\:\:\right.$$

The core idea of this module is to first divide the input features into several groups, then merge the current group’s subset $\:{X}_{i}$ with the previous output $\:{X}_{i-1}$ through residual connections. As $\:i$ increases, the receptive field also enlarges. Finally, the n groups of features are concatenated along the channel dimension and passed through a $\:1\times\:1$ convolution for feature fusion, producing the final output. This multi-scale feature extraction module often outperforms traditional branch-based feature extraction modules.

#### SE attention module

**Fig. 6:**
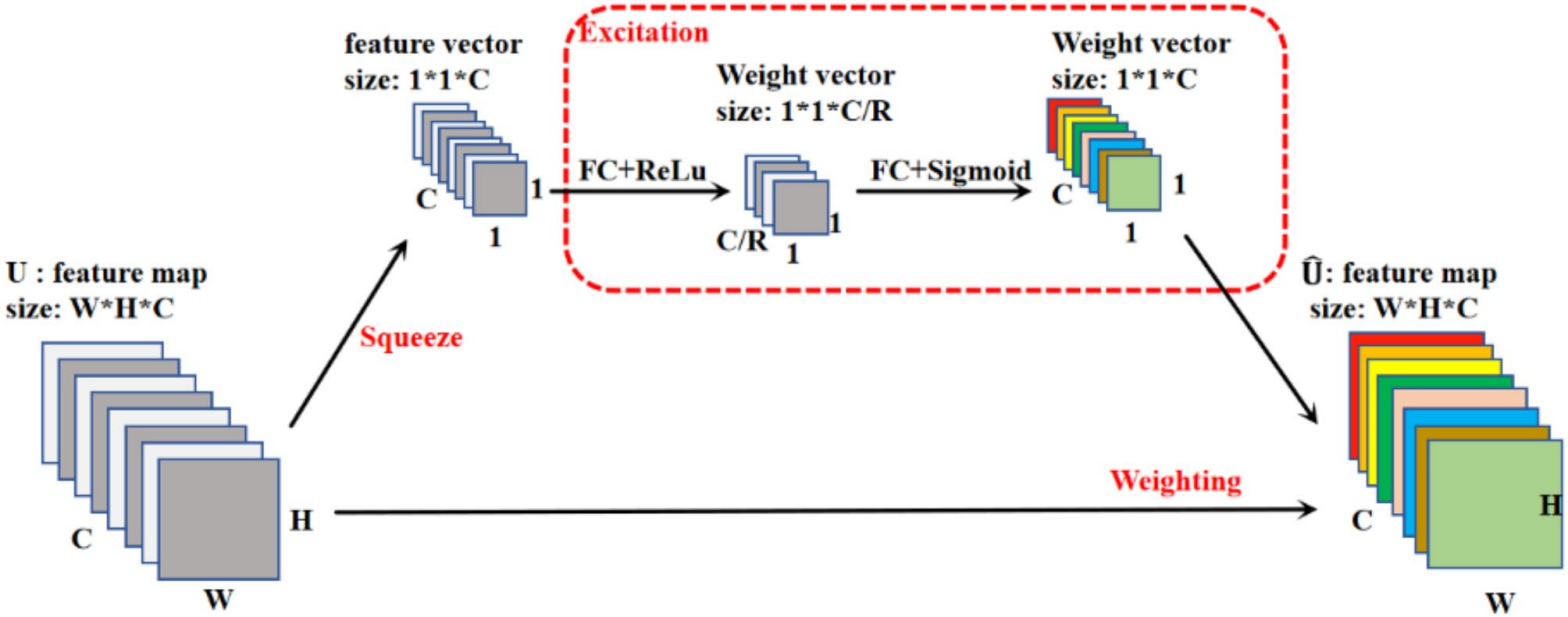
Squeeze and excitation module structure (SE)^31^.

SE attention module is a kind of channel attention module^32^. Weights are assigned according to the importance of different channels to bias the network towards more valuable information^31^. The main structure of the SE module is shown in Fig. 6, which can be divided into two steps: squeeze and excitation.

Squeeze operation is a compression process. The feature mapping with spatial dimension W*H is compressed into one dimension. This step is done by global average pooling. The dimensionality of the original feature map is reduced to 1*1. The purpose of this step is to obtain the global information of each channel. The squeeze operation can be expressed as:

$$\:{F}_{sq}\left({U}_{c}\right)=\frac{1}{H\text{*}W}\sum\:_{i=1}^{H}\sum\:_{j=1}^{W}{U}_{c}\left(i,j\right)$$

The excitation operation involves learning the weight parameters for each channel using two fully connected layers and activation functions, based on the results obtained from the preceding global pooling. The first fully connected layer changes the tensor’s dimensions from (1 * 1 * C) to (1 * 1 * C / R), where R is a hyperparameter. This step reduces the number of channels in the tensor to 1/R of the original number. The first fully connected layer uses ReLU as its activation function. The second fully connected layer restores the dimensions from (1 * 1 * C / R) back to the original (1 * 1 * C). The second fully connected layer uses the Sigmoid activation function, which maps the output values to a range between 0 and 1, representing the weight information for each channel. The purpose of this process is to learn weight parameters through the neural network, assigning different levels of importance or weight to different channels. ReLU and Sigmoid are commonly used activation functions, with ReLU increasing non-linearity and Sigmoid constraining the output between 0 and 1 to represent weight information.

Multiply these weights with the original feature map, i.e., weight the feature map for each channel. The weights of the different channels in the feature map are dynamically adjusted so that the network can focus more on features that are useful for the task. The more important feature channels will receive higher weights and be more focused on by the network. The dimensional size of the weighted feature map is the same as the original feature map, and the SE module does not change the small size of the feature map.

### Pyramid dilated convolution module

Dense dilated convolution has been widely applied in medical image segmentation tasks requiring fine segmentation^16–18,33,34^, such as precise segmentation of skin lesion boundaries. By introducing dense connections between convolutional layers, each layer’s input includes not only the output of the previous layer but also the outputs of all preceding layers. This dense connection method captures multi-level feature information more effectively, aiding in extracting richer features. By incorporating dilated convolutions with different dilation rates, the receptive field can be significantly expanded without increasing the number of parameters. This enables the model to focus on both local and global information, enhancing its ability to capture target boundaries and details.

To further improve the network’s feature extraction capabilities, this paper proposes a novel Pyramid Dilated Convolution (PDC) module. By setting different dilation rates, the module captures contextual information at multiple scales. This approach allows the network to obtain fine-grained information and expand the receptive field, thereby efficiently extracting more effective features and richer fine-grained information.

**Fig. 7:**
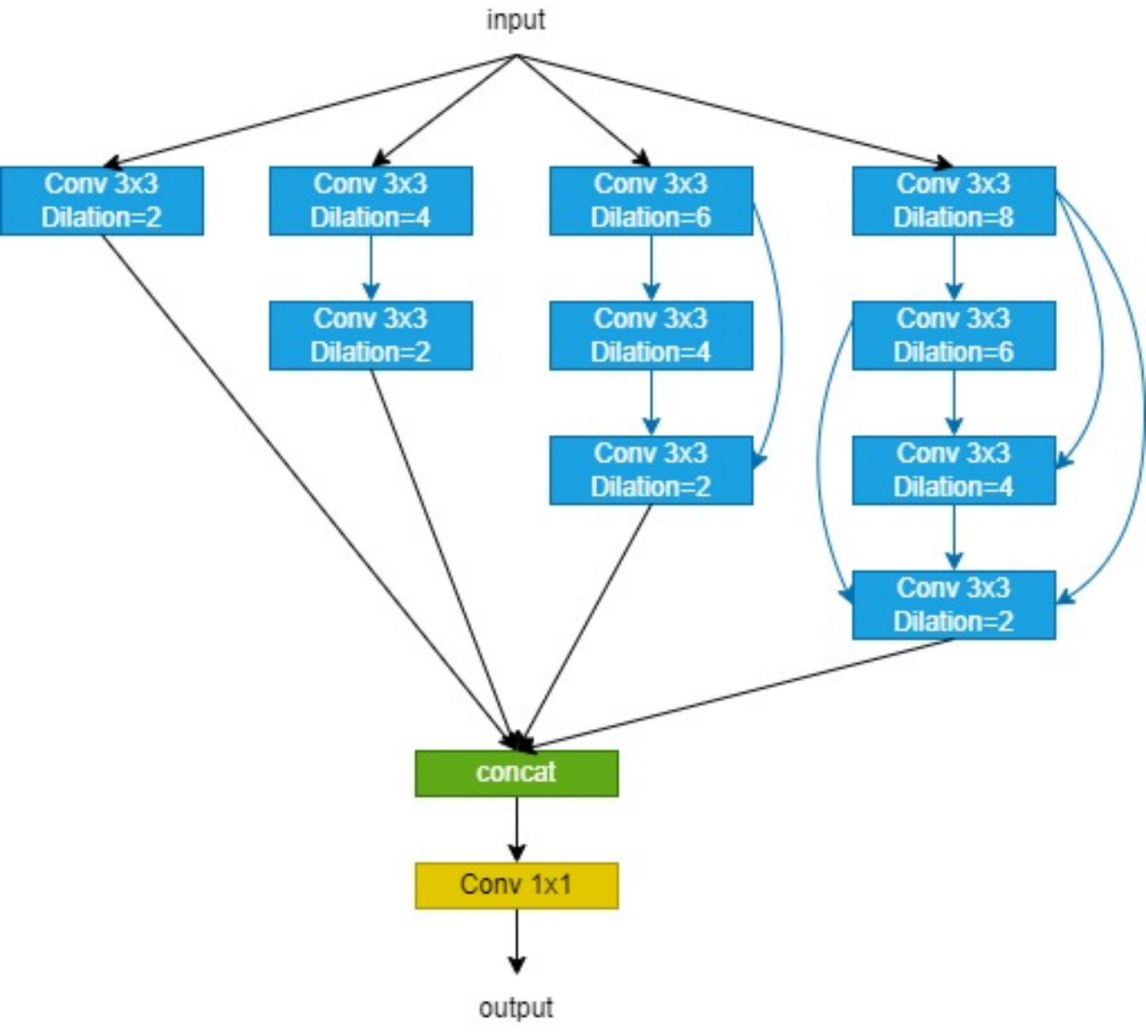
Structure of the pyramid dilated convolution (PDC) module.

The PDC block is shown in Fig. 7. Our proposed PDC block can capture broader and deeper semantic features by injecting four branches. Individual dilation convolutions on each branch are stacked in a cascade mode between them. It allows each feature map to undergo multiple dilation-convolution operations at different rates, thus capturing feature information at various scales. Finally, after the feature maps at each level are stitched together, a 1*1 convolution is performed to obtain the output. The performance of medical image segmentation is improved by combining the newly proposed PDC block trunk encoder-decoder structure to capture more abstract features and retain more spatial information. Usually, convolution with large receptive fields can extract and generate more abstract features for large objects, while convolution with small receptive fields is more suitable for small objects. By combining dilation convolution with different dilation rates, the PDC block can extract features for objects of various sizes.

## Materials and experiments

### Datasets

To validate the proposed method, extensive experiments were conducted on ISIC 2016^35^, ISIC 2017^36^、ISIC 2018^10^, PH2^37^, and HAM10000^38^. These skin images were collected from various devices across multiple international clinical institutions. The ISIC 2016 dataset contains 900 training images and 379 test images. The ISIC 2017 dataset includes 2,000 training images and 600 test images. The ISIC 2018 public dataset comprises 2596 images, which we randomly split into 2,096 images for training and 500 images for testing. Table 1 provides detailed information on the public datasets used in our experiments.

The preprocessing steps not only remove irrelevant information and reduce noise but also ensure consistency in subsequent processing, thereby enhancing the model’s performance. The original datasets provided by ISIC contain images with varying resolutions. First, we cropped the black borders surrounding some sample images to reduce noise interference. Then, all images were resized to a uniform input size (576 × 576 in our experiments).

**Table 1:** Detailed information of the public datasets used in the experiments.

Datasets	Year	Modality	Size	Train/ Validate/Test	Resolution
ISIC2016	2016	Dermoscopy	1279	900/-/379	Range from 566 × 679 to 2848 × 4288 pixels
ISIC 2017	2017	Dermoscopy	2750	2000/150/600	Range from 540 × 722 to 4499 × 6748 pixels
ISIC 2018	2018	Dermoscopy	3694	2594/100/1000	Range from 540 × 576 to 4499 × 6748 pixels
PH2	2013	Dermoscopy	200	-	Range from 553 × 763 to 577 × 769 pixels
HAM10000	2020	Dermoscopy	10,015	-	All images of 600 × 450 pixels

### Implementation details

The proposed model was implemented in PyTorch and run on an NVIDIA RTX 3090. We selected Adam as the optimizer for stochastic gradient descent (SGD) optimization, guided by the loss function. Cross-entropy was used as the loss function. The batch size was set to 16, with a maximum of 100 iterations. We used a batch normalization decay of 0.998 and a momentum of 0.9. The initial learning rate was 0.01, gradually decreasing to a final learning rate of 0.000001.

### Evaluation metrics

In the realm of segmenting skin lesion pictures, the commonly used six assessment metrics for performance include accuracy (ACC), specificity (SPE), sensitivity (SEN), (JI), recall, and dice coefficient(DC).

For evaluating segmentation performance in the ISIC challenges, the Jaccard Index was considered the most crucial metric. Accuracy (ACC) is the ratio of the number of samples correctly predicted by the model to the total number of samples. Specificity (SPE) measures the ability of the model to correctly predict a negative class in a negative class sample. Sensitivity (SEN) measures the ability of the model to correctly predict as positive classes in positive class samples. Dice coefficient (Dice) measures the similarity between the predicted region of an object and the ground truth region, and indicates the overlap ratio between the predicted segmentation results and the ground truth labels. The calculation formula for the above indicators can be expressed in Table 2.

**Table 2:** Evaluation metrics for segmentation performance.

Metrics	Formulas	Interpretation
Sensitivity	$\:\text{S}\text{e}\text{n}=\frac{\text{T}\text{P}}{\text{T}\text{P}+\text{F}\text{N}}=TPR$	The ratio of correctly classified samples to the total number of samples (all classes).
Specificity	$\:\text{S}\text{p}\text{e}=\frac{\text{T}\text{N}}{\text{T}\text{N}+\text{F}\text{P}}=TNR$	The ratio of correctly predicted positive samples to the total predicted positive samples.
Accuracy	$\:\text{A}\text{c}\text{c}=\frac{\text{T}\text{P}+\text{T}\text{N}}{\text{T}\text{P}+\text{T}\text{N}+\text{F}\text{P}+\text{F}\text{N}}$	The ratio of correctly predicted negative samples to the total actual negative samples.
Dice coefficient	$\:DC=\frac{2\text{*}\text{T}\text{P}}{2\text{*}\text{T}\text{P}+\text{F}\text{N}+\text{F}\text{P}}$	The Dice coefficient measures the similarity between predicted and actual segmentation areas.
Jaccard index	$\:JI=\frac{\text{T}\text{P}}{\text{T}\text{P}+\text{F}\text{N}+\text{F}\text{P}}$	The Jaccard index is used to assess the degree of overlap between the predicted segmented region and the actual region.

Performance evaluation of skin lesion segmentation methods involves comparing their outputs against the ground truth images. Each pixel can be classified into four categories: TP (True Positive, correctly identified lesion pixels), FP (False Positive, non-lesion pixels incorrectly classified as lesion), FN (False Negative, lesion pixels incorrectly classified as non-lesion), and TN (True Negative, correctly identified non-lesion pixels).

The importance of the above metrics varies slightly in different segmentation tasks. A single evaluation metric is often not comprehensive enough to evaluate the segmentation quality of an algorithm. In real segmentation tasks, we usually use multiple metrics to evaluate the segmentation performance of the algorithm more scientifically and reasonably.

## Results

### Comparative with different datasets

In this section, we compare the quantitative and qualitative analyses of the proposed MRP-UNet network on several public datasets, including ISIC2016, 2017, 2018, PH2, and HAM10000, respectively.

#### Quantitative analysis

Table 3 presents the quantitative results of the proposed MRP-UNet method on five different datasets: ISIC 2016, ISIC 2017, ISIC 2018, PH2, and HAM10000. The performance metrics considered include Accuracy (Acc), Sensitivity (Sen), Specificity (Spe), Jaccard Index (JI), and Dice Coefficient (DC).

The results demonstrate that the MRP-UNet achieves consistently high performance across all datasets. Notably, the model attains an accuracy of 96.17% on the ISIC 2016 dataset and 96.13% on the PH2 dataset. Sensitivity values are also impressive, with the highest being 92.27% on the ISIC 2016 dataset and 91.92% on the PH2 dataset, indicating the model’s robustness in detecting true positives.

The specificity metrics further affirm the model’s capability in identifying true negatives, with the highest value of 93.44% on the ISIC 2018 dataset and a notable 93.17% on the HAM10000 dataset. Additionally, the Jaccard Index (JI) and Dice Coefficient (DC) are crucial for assessing the overlap between the predicted and actual segmented regions. The JI scores range from 90.18 to 92.41%, and the DC scores range from 91.98 to 94.19%, highlighting the model’s accuracy and consistency in segmentation tasks.

Overall, these results underline the effectiveness of the proposed MRP-UNet method in skin lesion segmentation tasks, demonstrating superior performance across multiple public datasets.

**Table 3:** Quantitative results for the ISIC2016,ISIC2017,ISIC2018,PH2,HAM10000 dataset using the proposed MRP-UNet.

Method	Dataset	Acc	Sen	Spe	JI	DC
MRP-UNet (Proposed)	ISIC 2016	96.17	92.27	92.37	92.01	91.98
MRP-UNet (Proposed)	ISIC2017	93.51	90.42	92.18	92.41	93.14
MRP-UNet (Proposed)	ISIC2018	95.51	88.57	93.44	91.28	92.36
MRP-UNet (Proposed)	PH2	96.13	91.92	92.65	90.77	94.19
MRP-UNet (Proposed)	HAM10000	94.64	89.85	93.17	90.18	92.95

#### Qualitative analyses

**Fig. 8:**
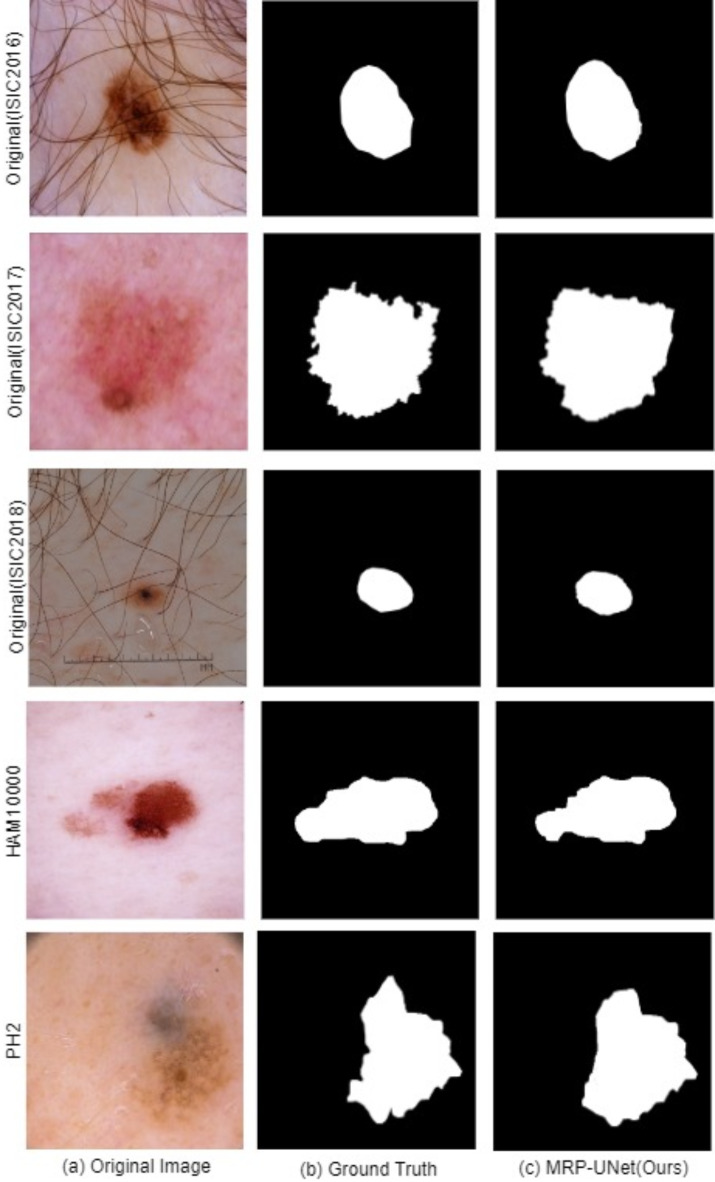
visual qualitative comparison of MRP-UNet on ISIC 2016, ISIC 2017, ISIC 2018, PH2, and HAM10000 Datasets. (**a**) Original image. (**b**) Ground truth (**c**) MRP-UNet (Ours).

In the case illustrated in Fig. 8, we can observe several challenging scenarios, such as lesions appearing at different scales, with varying color distributions, and at different locations on the body. Figure 8 shows the visual qualitative comparison of MRP-UNet on the ISIC 2016, ISIC 2017, ISIC 2018, PH2, and HAM10000 datasets. The figure shows that MRP-UNet often provides segmentation results that are close to the ground truth. The proposed method can accurately segment boundaries, especially when interfering and misleading factors are in the background. For situations where the lesions are very similar to the surrounding skin, MRP-UNet also achieves better results with smaller errors.

### Comparative with SOTA

In this section, we compare the quantitative analysis of the proposed MRP-UNet network with other SOTA skin lesion segmentation methods including U-Net^15^, DAGAN^18^, AMCC-Net^39^, MSCA-Net^40^, TMAHU-Net^41^, FDUM-Net^42^, UCM-Net^43^, MHorUNet^44^.

Tables 4, 5, 6, 7 and 8 present a quantitative comparison of MRP-UNet with state-of-the-art (SOTA) skin lesion segmentation methods across five different public datasets. The metrics evaluated include accuracy (ACC), sensitivity (Sen), specificity (Spe), Jaccard Index (JI), and Dice Coefficient (DC). This indicates that our multiscale input fusion architecture, residual attention mechanism, and pyramid dilated convolution modules are highly effective in improving skin lesion segmentation.

The performance of the proposed MRP-UNet is comprehensively compared against several state-of-the-art (SOTA) methods, including U-Net, DAGAN, AMCC-Net, MSCA-Net, TMAHU-Net, FDUM-Net, UCM-Net, and MHorUNet across five datasets: ISIC 2016, ISIC 2017, ISIC 2018, PH2, and HAM10000. The evaluated metrics include accuracy (Acc), sensitivity (Sen), specificity (Spe), Jaccard Index (JI), and Dice Coefficient (DC).

On the ISIC 2016 dataset, MRP-UNet achieves the highest accuracy (96.17%), Jaccard Index (92.01%), and Dice Coefficient (91.98%) compared to the other models. The improvement over DAGAN (which achieved 94.85% Acc) is notable, especially in terms of JI and DC, where MRP-UNet exhibits a substantial performance gain. This indicates that MRP-UNet’s combination of MIF, Res2-SE, and PDC modules allows for better handling of complex lesion boundaries and multi-scale features. The specificity (92.37%) of MRP-UNet also outperforms other methods, suggesting that it is better at avoiding false positives. This is particularly important in medical image analysis, where over-segmentation could lead to incorrect diagnoses.

The ISIC 2017 dataset poses more challenging cases due to blurred and irregular lesion boundaries. MRP-UNet achieves the highest accuracy (93.51%), Jaccard Index (92.41%), and Dice Coefficient (93.14%), outperforming AMCC-Net, FDUM-Net, and other SOTA methods. Particularly, MRP-UNet significantly improves over DAGAN, which achieved 89.63% JI and 88.64% DC, demonstrating the effectiveness of the multi-scale and attention mechanisms in capturing fine-grained lesion details. The sensitivity (90.42%) of MRP-UNet is also higher than other methods, which is crucial for detecting all lesion regions, including the challenging edge areas. This indicates that MRP-UNet minimizes under-segmentation compared to other methods like TMAHU-Net and UCM-Net, which show lower sensitivity.

In the ISIC 2018 dataset, MRP-UNet again surpasses all other methods, with the highest accuracy (95.51%), Jaccard Index (91.28%), and Dice Coefficient (92.36%). While DAGAN and FDUM-Net also perform well (with DAGAN achieving 93.04% Acc and FDUM-Net achieving 94.28% Acc), MRP-UNet’s improved performance on the Jaccard Index and Dice Coefficient metrics highlights its superior ability to accurately segment both large and small lesions with varying shapes. The specificity (93.44%) of MRP-UNet is particularly strong in this dataset, which means it effectively discriminates between the lesion and the surrounding healthy skin. This high specificity suggests fewer false positives, further enhancing its clinical applicability.

On the PH2 dataset, MRP-UNet achieves 96.13% accuracy, outperforming all other methods, including FDUM-Net (93.83% Acc) and DAGAN (92.14% Acc). Notably, MRP-UNet also achieves the highest Jaccard Index (90.77%) and Dice Coefficient (94.19%), showcasing its capability to produce precise segmentation even in cases with weak or blurred boundaries. The combination of high accuracy, sensitivity (91.92%), and specificity (92.65%) reflects the model’s balanced performance. It excels in identifying the lesion area while maintaining strong generalization to new samples, which is critical for real-world medical applications.

In the HAM10000 dataset, MRP-UNet shows its robustness by achieving the highest accuracy (94.64%), Jaccard Index (91.18%), and Dice Coefficient (92.95%), outperforming models like AMCC-Net and FDUM-Net. Given the larger variability in lesion types and sizes in HAM10000, MRP-UNet’s superior performance demonstrates its ability to generalize across a diverse range of lesion characteristics. The model also shows strong specificity (93.17%), indicating a lower rate of false positives compared to UCM-Net and MSCA-Net. This is vital for reducing unnecessary clinical follow-ups or interventions.

**Table 4:** Qualitative analysis of the proposed method and SOTA methods on datasets ISIC2016.

Reference	Method (Year)	Acc	Sen	Spe	JI	DC
^15^	U-Net (2015)	86.26	81.62	84.98	85.81	83.69
^18^	DAGAN (2020)	94.85	91.56	**93.21**	91.52	83.16
^39^	AMCC-Net (2023)	88.26	87.32	91.23	90.14	86.84
^40^	MSCA-Net (2023)	87.22	89.01	86.41	87.04	85.68
^41^	TMAHU-Net (2024)	93.73	85.91	90.94	89.63	89.24
^42^	FDUM-Net (2024)	94.11	90.10	89.52	87.88	91.52
^43^	UCM-Net (2024)	90.67	89.43	85.63	87.29	86.43
^44^	MHorUNet (2024)	87.67	88.133	87.30	86.43	88.93
Proposed (Ours)	MRP-UNet (2024)	**96.17**	**92.27**	92.37	**92.01**	**91.98**

Bold text indicates the best result in each metric.

**Table 5:** Qualitative analysis of the proposed method and SOTA methods on datasets ISIC2017.

Reference	Method (Year)	Acc	Sen	Spe	JI	DC
^15^	U-Net (2015)	85.12	83.32	86.61	84.96	84.04
^18^	DAGAN (2020)	92.62	88.98	90.75	89.63	88.64
^39^	AMCC-Net (2023)	87.14	89.93	87.73	90.12	90.77
^40^	MSCA-Net (2023)	89.13	87.53	86.98	89.11	88.98
^41^	TMAHU-Net (2024)	90.81	86.38	92.29	86.46	89.73
^42^	FDUM-Net (2024)	92.45	89.54	**93.00**	87.13	89.15
^43^	UCM-Net (2024)	87.54	88.65	90.12	85.67	89.12
^44^	MHorUNet (2024)	88.58	87.83	88.79	86.04	85.55
Proposed (Ours)	MRP-UNet (2024)	**93.51**	**90.42**	92.18	**92.41**	**93.14**

Bold text indicates the best result in each metric.

**Table 6:** Qualitative analysis of the proposed method and SOTA methods on datasets ISIC2018.

Reference	Method (Year)	Acc	Sen	Spe	JI	DC
^15^	U-Net (2015)	86.37	80.52	84.26	82.43	85.64
^18^	DAGAN (2020)	93.04	89.75	92.58	90.19	87.64
^39^	AMCC-Net (2023)	87.38	**90.25**	88.45	88.38	90.28
^40^	MSCA-Net (2023)	86.76	88.65	89.72	87.28	88.63
^41^	TMAHU-Net (2024)	92.12	84.46	93.01	87.86	88.44
^42^	FDUM-Net (2024)	94.28	88.52	91.56	86.79	90.45
^43^	UCM-Net (2024)	89.01	87.90	88.68	86.41	86.67
^44^	MHorUNet (2024)	88.49	87.59	86.43	89.04	87.54
Proposed (Ours)	MRP-UNet (2024)	**95.51**	88.57	**93.44**	**91.28**	**92.36**

Bold text indicates the best result in each metric.

**Table 7:** Qualitative analysis of the proposed method and SOTA methods on datasets PH2.

Reference	Method (Year)	Acc	Sen	Spe	JI	DC
^15^	U-Net (2015)	85.66	83.41	83.42	83.62	85.17
^18^	DAGAN (2020)	92.14	87.16	89.01	88.63	89.55
^39^	AMCC-Net (2023)	88.62	87.63	90.69	87.19	88.91
^40^	MSCA-Net (2023)	88.34	86.16	88.31	87.43	87.27
^41^	TMAHU-Net (2024)	91.42	88.73	91.89	86.92	88.33
^42^	FDUM-Net (2024)	93.83	85.63	89.18	89.34	91.74
^43^	UCM-Net (2024)	89.42	88.97	90.73	86.42	88.18
^44^	MHorUNet (2024)	87.93	89.62	88.51	87.13	86.83
Proposed (Ours)	MRP-UNet (2024)	**96.13**	**91.92**	**92.65**	**90.77**	**94.19**

Bold text indicates the best result in each metric.

**Table 8:** Qualitative analysis of the proposed method and SOTA methods on datasets HAM10000.

Reference	Method (Year)	Acc	Sen	Spe	JI	DC
^15^	U-Net (2015)	85.62	83.12	84.17	86.94	82.64
^18^	DAGAN (2020)	91.52	88.61	92.72	89.74	88.52
^39^	AMCC-Net (2023)	87.64	86.69	89.71	87.85	91.72
^40^	MSCA-Net (2023)	86.19	87.66	87.62	87.94	88.37
^41^	TMAHU-Net (2024)	87.64	88.84	89.63	89.74	89.55
^42^	FDUM-Net (2024)	90.53	88.11	91.73	88.45	89.81
^43^	UCM-Net (2024)	88.83	87.91	90.64	87.62	90.15
^44^	MHorUNet (2024)	90.12	87.29	86.23	88.35	90.73
Proposed (Ours)	MRP-UNet (2024)	**94.64**	**89.85**	**93.17**	**91.18**	**92.95**

Bold text indicates the best result in each metric.

**Fig. 9:**
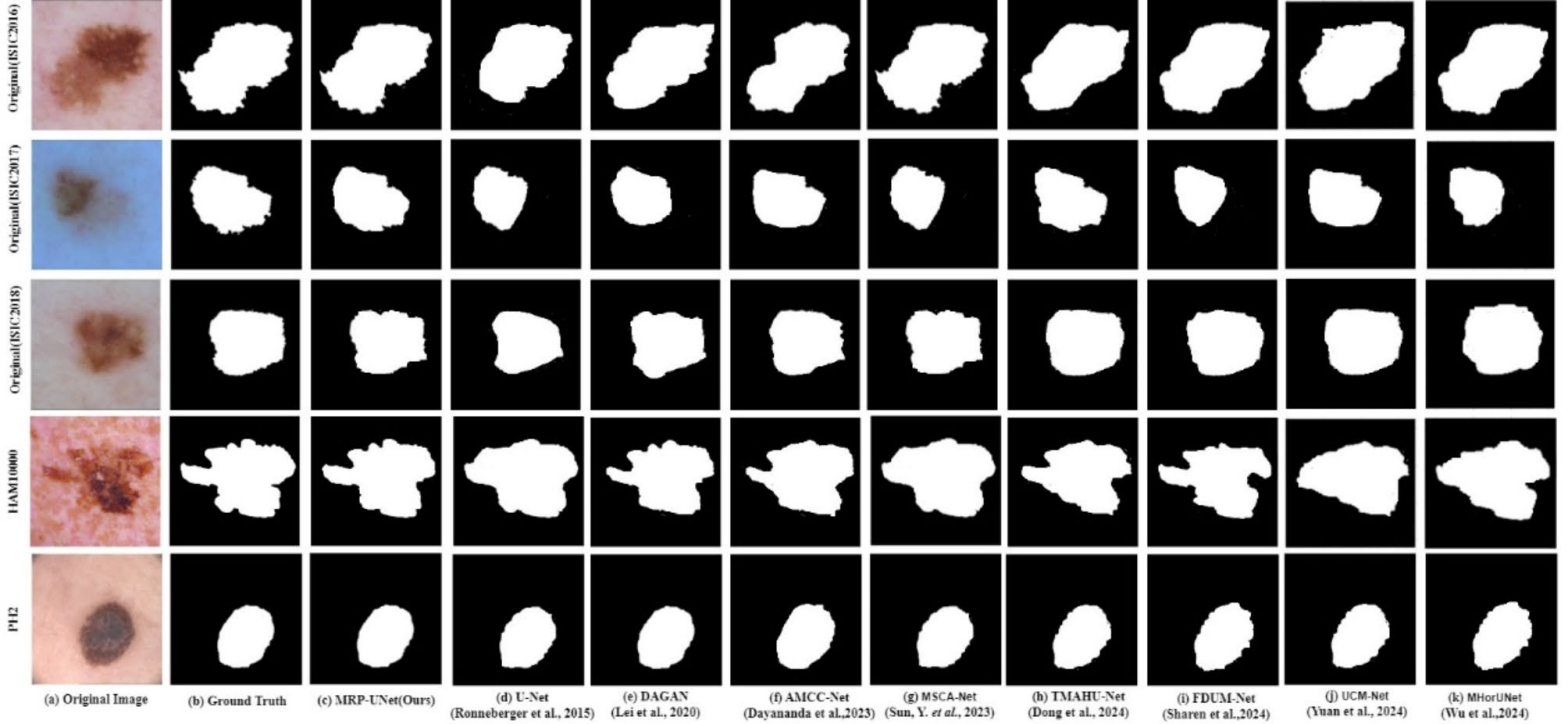
Visual qualitative comparison With SOTA method on ISIC 2016, ISIC 2017, ISIC 2018, PH2, and HAM10000 datasets. (**a**) Original image. (**b**) Ground truth (**c**) MRP-UNet (Ours). (**d**) U-Net. (**e**) DAGAN. (**f**) AMCC-Net. (**g**) MSCA-Net. (**h**) TMAHU-Net. (**i**) FDUM-Net. (**j**) UCM-Net. (**k**) MHorUNet.

Figure 9 shows a visual comparison of various skin lesion segmentation methods across five different datasets: ISIC 2016, ISIC 2017, ISIC 2018, HAM10000, and P12. Each row represents a skin lesion image, and each column displays the results of six segmentation models: MRP-UNet (our model), U-Net, DAGAN, AMCC-Net, TMAHU-Net, and FDUM-Net. The segmentation results of our proposed MRP-UNet are the closest to the ground truth across different images, demonstrating its robustness in handling variations in lesion appearance. It outperforms other methods in accurately segmenting the overall contour of the lesion and capturing edge details.

In comparison, U-Net generally provides good segmentation but sometimes fails to capture details or irregular boundaries; DAGAN may over-segment or under-segment in some areas; AMCC-Net’s segmentation results are smooth and accurate but may struggle with very irregular shapes; TMAHU-Net excels in preserving lesion structures; and FDUM-Net captures complex boundaries but occasionally introduces slight segmentation errors. Overall, this comparative analysis highlights the superior performance of MRP-UNet in various challenging scenarios, aiding in understanding the effectiveness of different segmentation methods in skin lesion analysis.

### Ablation experiment

#### Experiment strategy

To validate the effectiveness of the proposed individual modules, a series of ablation analyses are performed on the PH2 dataset. We set the conventional U-Net as a benchmark. The detailed configurations of the modules are as follows:

Module 1 (baseline): U-Net as a benchmark.

Module 2 (with MIF): The baseline Module with the addition of the MIF module.

Module 3 (with Res2Net): The baseline Module with the addition of the Res2-SE module.

Module 4 (with SE): The baseline Module with the addition of the Res2-SE module.

Module 5 (with Res2-SE): The baseline Module with the addition of the Res2-SE module.

Module 6 (with PDC): The baseline module with the addition of the PDC module.

Module 7 (with MIF and Res2-SE): The baseline module with both the MIF and Res2-SE modules.

Module 8 (with MIF and PDC): The baseline module with both the MIF and PDC modules.

Module 9 (with Res2-SE and PDC): The baseline module with both the Res2-SE and PDC modules.

Module 10 (with MIF, Res2-SE and PDC): The baseline module with the full combination of MIF, Res2-SE, and PDC modules, corresponding to the complete MRP-UNet.

All modules follow the same training strategy and hyperparameters to ensure a consistent evaluation. The performance of each module is compared across key metrics, and the results demonstrate the individual and combined contributions of the MIF, Res2-SE, and PDC modules to the overall performance of MRP-UNet.

#### Quantitative analysis

The combination of all three modules (MIF, Res2-SE, and PDC) in MRP-UNet delivers the best performance across all evaluation metrics, with an accuracy of 96.13%, significantly surpassing all other models. This demonstrates the complementary nature of these modules, where their combined effect improves feature extraction and multi-scale handling, leading to superior segmentation capabilities. Compared to individual modules, MRP-UNet shows statistically significant overall performance improvement.

**Fig. 10:**
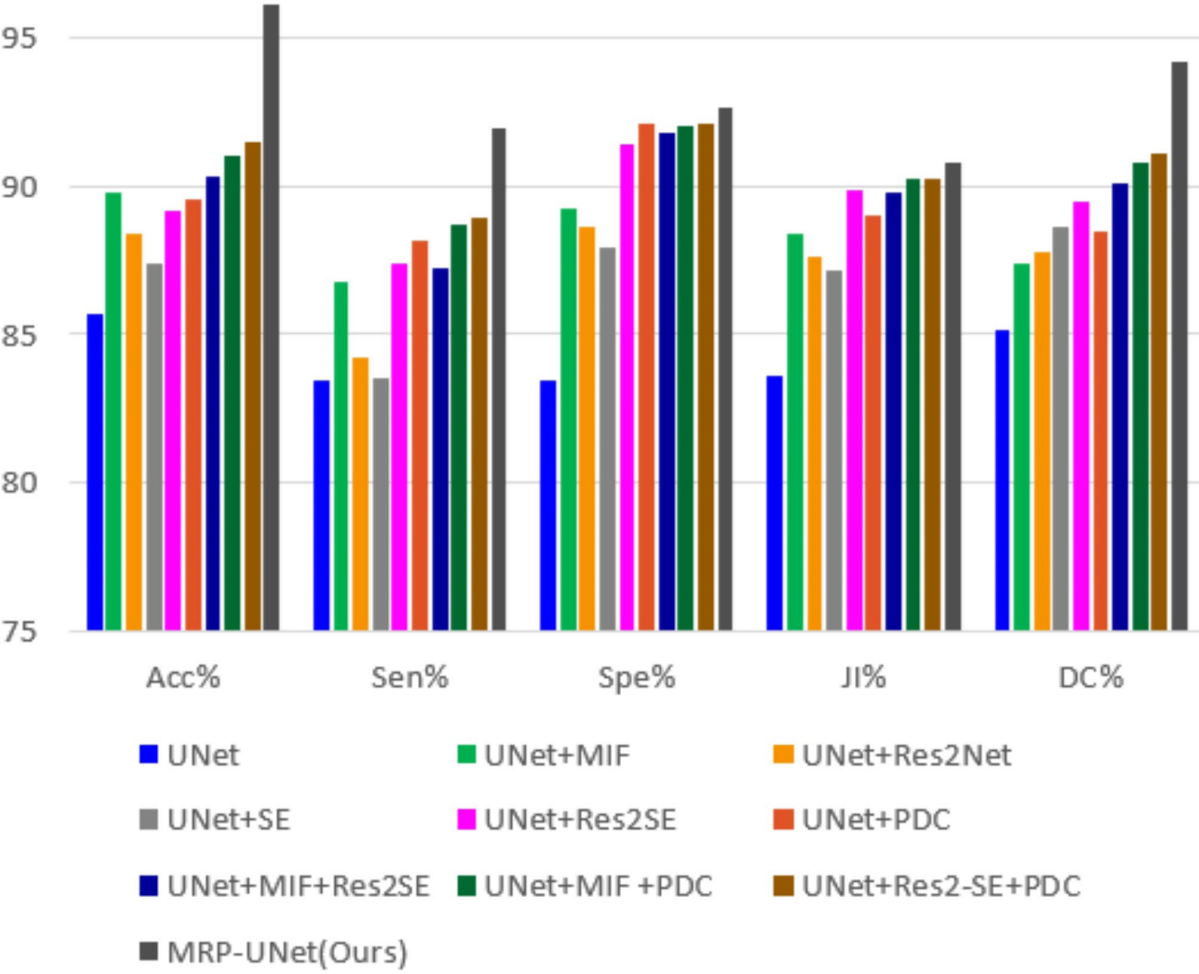
Ablation study for the proposed MRP-UNet.

Statistical comparisons were performed in the PH2 data set, as shown in Table 9. The ablation study results demonstrate that each of the MIF, Res2-SE, and PDC modules contributes significantly to skin lesion segmentation. When combined in MRP-UNet, they achieve the highest overall performance, validating the effectiveness of the proposed modules in enhancing segmentation accuracy. Figure 10 illustrates the results more visually with bar charts. It can be seen that the proposed method achieves better performance. These results validate the effectiveness of the state-of-the-art of modules in the MRP-UNet network.

**Table 9:** Quantitative results of ablation experiments on the PH2 dataset using the proposed MRP-UNet.

Method	Acc	Sen	Spe	JI	DC
UNet	85.66	83.41	83.42	83.62	85.17
UNet + MIF	89.74	86.73	89.25	88.41	87.39
UNet + Res2Net	88.35	84.23	88.61	87.63	87.74
UNet + SE	87.41	83.53	87.89	87.18	88.64
UNet + Res2SE	89.12	87.41	91.40	89.85	89.49
UNet + PDC	89.55	88.13	92.11	89.01	88.49
UNet + MIF + Res2SE	90.35	87.21	91.82	89.75	90.11
UNet + MIF + PDC	91.02	88.67	92.03	90.25	90.78
UNet + Res2-SE + PDC	91.44	88.91	92.56	90.67	91.05
MRP-UNet (Ours)	**96.13**	**91.92**	**92.65**	**90.77**	**94.19**

Significant values are in bold.

#### Effectiveness of the MIF module

The MIF (Multi-Scale Input) module constructs multi-scale inputs using image pyramids, enhancing the model’s ability to handle lesions of varying sizes. This module is particularly effective in addressing scale variation in skin lesions, significantly improving segmentation accuracy. Experimental results show that adding the MIF module leads to a notable performance boost, such as the accuracy of UNet + MIF reaching 89.74%, compared to the baseline UNet’s 85.66%.

#### Effectiveness of the Res2-SE module

The Res2-SE module combines the Res2Net architecture with the SE (Squeeze-and-Excitation) attention mechanism, replacing the original 3 × 3 convolution in the encoder of U-Net. This module enhances feature extraction by focusing on important regions, improving the model’s overall segmentation performance by emphasizing key channel features. After adding the Res2-SE module, significant improvements were observed across all metrics, especially in specificity (Spe) and Dice Coefficient (DC), indicating its effectiveness in extracting critical features.

#### Effectiveness of the PDC module

The PDC (Pyramid Dilated Convolution) module introduces multi-scale dilated convolutions between the encoder and decoder. By merging feature maps from different dilation rates, it captures richer multi-scale features. Results show that the PDC module substantially improves model performance, particularly in accuracy (Acc) and specificity (Spe). For instance, UNet + PDC achieved a specificity of 92.11%, outperforming other single-module configurations.

## Limitations and future work

**Fig. 11:**
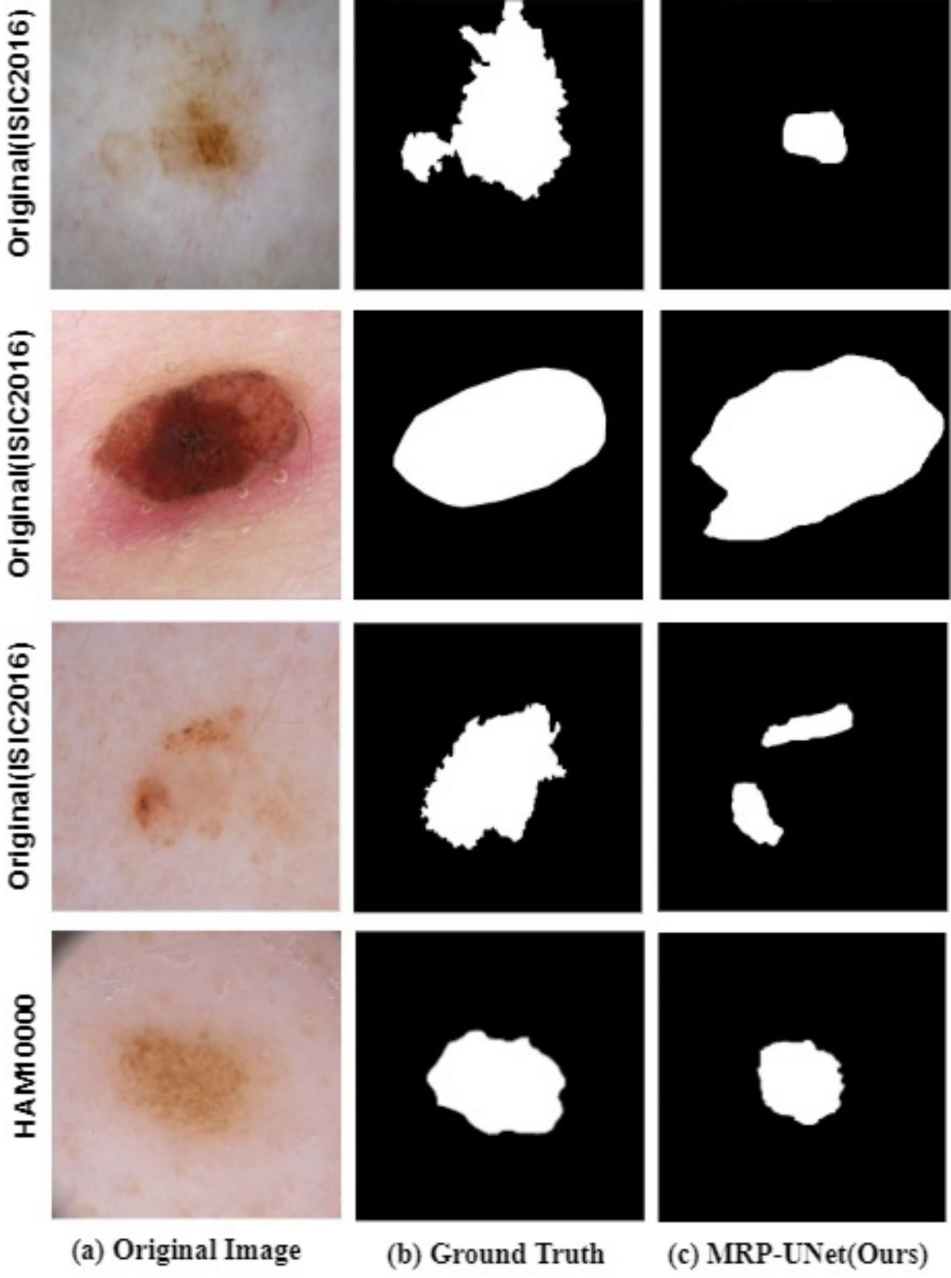
Examples of failure cases with our proposed method on the ISIC 2016 and HAM10000 datasets. The first three rows are from ISIC 2016, and the fourth row is from HAM10000. The first column shows the original images, the second column shows the ground truth, and the third column shows the segmentation results using our method.

In the experimental part of this paper, we demonstrate that MRP-UNet outperforms mainstream methods on five publicly available datasets. However, this work still has some limitations. First, there are still instances where our proposed method struggles to segment images with fuzzy edges and weak feature information, as demonstrated in Fig. 11. The model may misjudge lesion boundaries while identifying the overall contours of the lesion area, indicating that edge rationalization remains a challenge. Secondly, our method is a data-driven approach, meaning it relies on large datasets annotated by physicians to achieve optimal results. This creates a dependency on labeled datasets, which may limit its applicability in scenarios with limited annotated data. Finally, MRP-UNet is currently tailored for skin lesion segmentation and may encounter challenges when applied to other medical image segmentation tasks due to domain-specific variations.

To address these limitations, future research will focus on several key areas. First, we will explore combining Transformer and GAN models with U-Net to improve segmentation, particularly for images with low contrast and blurred boundaries. In addition, to reduce dependency on large labeled datasets, we will investigate weakly supervised learning approaches that can leverage minimal labeling while still delivering high-quality segmentation results. We aim to contribute to skin cancer diagnosis and enhance clinical practice. Furthermore, we plan to extend the generalizability of the proposed model to other medical domains, improving its application to a broader range of medical image segmentation tasks. By further refining and expanding the functionality of MRP-UNet, we hope to explore its potential across various fields in medical imaging.

## Conclusion

Early detection of skin lesions is essential for treatment, but existing methods often struggle with accurate boundary segmentation. To address this, we propose MRP-UNet, a U-shaped network with MIF, Res2-SE, and PDC modules to enhance feature extraction and segmentation accuracy. Experiments on five public datasets confirm the effectiveness of MRP-UNet, achieving superior performance across challenging cases.

However, despite the promising results, some limitations exist in our current work. MRP-UNet struggles with segmenting images with unclear boundaries and weak features. Additionally, the method’s reliance on large annotated datasets restricts its use in situations with limited labeled data. Future work will address these limitations by incorporating advanced techniques such as Transformer and GAN models, as well as exploring weakly supervised approaches to reduce reliance on labeled datasets. We also aim to extend the model’s application to other medical image segmentation tasks, enhancing its generalizability across different medical fields.

## Data Availability

The datasets generated during and analyzed during the current study are available in the public repository, [https://challenge.isic-archive.com/data/;https://www.fc.up.pt/addi/ph2%20database.html; https://www.kaggle.com/datasets/kmader/skin-cancer-mnist-ham10000]
